# Thermodynamic Investigation and Mixed Ligand Complex Formation of 1,4-Bis-(3-aminopropyl)-piperazine and Biorelevant Ligands

**DOI:** 10.1155/2012/984291

**Published:** 2012-11-26

**Authors:** Ahmed A. El-Sherif, Mohamed R. Shehata, Mohamed M. Shoukry, Mohammad H. Barakat

**Affiliations:** Department of Chemistry, Faculty of Science, Cairo University, Giza 12613, Egypt

## Abstract

Thermodynamic parameters for protonation of 1,4-bis(3-aminopropyl)-piperazine (BAPP) and its metal complexation with some divalent metal ions were determined in aqueous solution at constant ionic strength (0.1 M NaNO_3_) using a potentiometric technique. The order of –ΔG^0^ and –ΔH^0^ was found to obey Co^2+^ < Ni^2+^ < Cu^2+^ > Zn^2+^, in accordance with the Irving-Williams order. The formation equilibria of zinc (II) complexes and the ternary complexes Zn(BAPP)L, where L = amino acid, amides, or DNA constituents), have been investigated. Ternary complexes are formed by a simultaneous mechanism. The concentration distribution of the complexes in solution was evaluated as a function of pH. Stoichiometry and stability constants for the complexes formed are reported and discussed. The stability of ternary complexes was quantitatively compared with their corresponding binary complexes in terms of the parameter Δlog K.

## 1. Introduction

Metal complexes of biologically important ligands are sometimes more effective than free ligands [1]. It is not surprising, therefore, that many authors have studied the coordination compounds of several central atoms. Mixed ligand complexes have a key role in biological chemistry [2] because the mixed chelation occurs commonly in biological fluids as millions of potential ligands are likely to compete for metal ions in vivo [3]. These create specific structures [4] and have been implicated in the storage and transport of active substances through membranes. Among these ligands are piperazine and its derivatives. Some piperazine derivatives were found to inhibit acute human immune deficiency HIV virus from chronically and latently infected cells containing proviral DNA [5]. Also the antimalarial activities of piperazine derivatives are also known [6]. The investigation of metal complexes of piperazine compounds will support their biological activity. The study of ternary complexes of transition metal ions with amino acids, peptides, or DNA units has been the focus of increasing research effort [7–10], which has revealed the role of metal ions at the molecular level. These types of complexes are implicated in the storage and transport of metal ions and of active substances through membranes. So, it is worthwhile to assemble information on their formation, stability, and structure and on the mutual influence of two ligands bound to the same metal ion. Zinc(II), among other transition metal ions, plays a vital role in biological processes. Zinc deficiency can cause unusual disorders in the development of the body, disorders in the metabolic system and prostate gland, and can result in mental retardation. Studies on model complexes of zinc(II) ions have focused to improve the understanding of the structure-reactivity relationship of the active site in zinc-enzymes [11–14]. In some of the model complexes the chelating ligands (e.g., polyamines) have been selected to bind to three or four coordination sites of Zn^II^ via N-donor atoms, with the next sites being occupied by other ligands [15–18].

In view of the above facts and in continuation of our published work on the formation equilibria of amino acids [19, 20], amides [21, 22], and DNA units [23, 24], we report herein the protonation constants of the free ligand (BAPP) and the stepwise stability constants for its complexes with a number of 3d divalent metal ions (Zn^2+^, Co^2+^, Ni^2+^, and Cu^2+^) and the thermodynamics of these systems. This was done through calculation of stability constants for their complexes at different temperatures. This work is also extended to present some correlations between the thermodynamic functions and some of well-known properties of the metal ions. Ternary complexes involving Zn(II) ion, BAPP and amino acid, peptide, or DNA constituents are investigated.

## 2. Experimental

### 2.1. Materials and Reagents

**Scheme 1:**
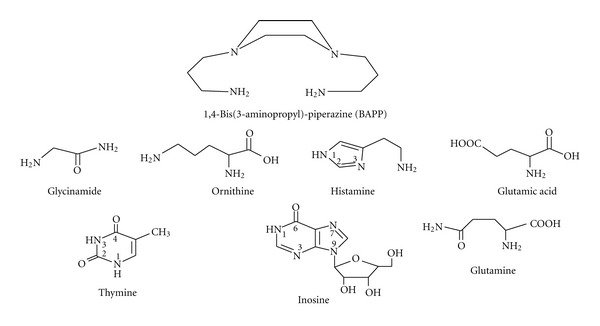
Structural formulae of some important investigated ligands.

1,4-Bis(3-aminopropyl)-piperazine (BAPP), Ni(NO_3_)_2_·6H_2_O, and Co(NO_3_)_2_·6H_2_O were provided from Aldrich Chem. Co. Cu(NO_3_)_2_·3H_2_O was provided from Fluka. Zn(NO_3_)_2_·6H_2_O was provided from FSA supplies. The metal ion solutions were prepared by dissolving A.R. grade metal salts in deionized water. Amino acids, peptides, and DNA constituents investigated are: alanine, threonine, serine, ornithine, glutamic acid, histamine·2HCl, glycinamide, glutamine, inosine, thymidine, and thymine. These materials were provided by Sigma Chem. Company and used without further purification. All solutions of the above reagents were freshly prepared in deionized water. All other chemicals used were of A.R. grade quality. Carbonate-free NaOH (titrant) was prepared and standardized against potassium hydrogen phthalate solution. The structural formula of BAPP and some selected biorelevant ligands are given in Scheme 1.

### 2.2. Apparatus and Procedures

 Potentiometric measurements were made using a Metrohm 686 titroprocessor equipped with a 665 Dosimat (Switzerland—Herisau). A thermostatted glass-cell was used and equipped with a magnetic stirring system, a Metrohm glass electrode, a thermometric probe, a microburettel delivery tube, and a salt bridge connected with the reference cell filled with 0.1 mol·dm^−3^ KCl solution in which saturated calomel electrode was dipped. The titroprocessor and electrode were calibrated daily with standard buffer solutions prepared according to NBS specifications at 25.0 ± 0.1°C [25] and I = 0.1 mol·dm^−3^, potassium hydrogen phthalate (pH 4.008), and a mixture of KH_2_PO_4_ and Na_2_HPO_4_ (pH 6.865). The BAPP solution was prepared in the protonated form by dissolving in HNO_3_ solution. The protonation constant of the ligand was determined potentiometrically by titrating the ligand (40 cm^3^) solution (1.25 × 10^−3^ mol·dm^−3^) of constant ionic strength 0.1 mol·dm^−3^, (adjusted with NaNO_3_). The stability constants of the binary complexes were determined by titrating 40 cm^3^ of a solution mixture of Zn^II^, Cu^II^, Co^II^, or Ni^II^ (1.25 × 10^−3^ mol·dm^−3^), the ligand (2.5 × 10^−3^ mol·dm^−3^), and NaNO_3_ (0.1 mol·dm^−3^). The stability constants of the ternary complexes were determined by titrating 40 cm^3^ of a solution mixture of Zn^II^ (1.25 × 10^−3^ mol·dm^−3^), the BAPP (1.25 × 10^−3^ mol·dm^−3^), the ligand (1.25 × 10^−3^ mol·dm^−3^), and NaNO_3_ (0.1 mol·dm^−3^). All titrations were performed in a purified nitrogen atmosphere, using aqueous 0.05 M NaOH as titrant. The pH meter readings were converted into hydrogen ion concentration by titrating HCl solution (0.05 mol·dm^−3^) with NaOH solution (0.05 mol·dm^−3^) at 25°C and I = 0.1 mol·dm^−3^ NaNO_3_. The pH is plotted against p[H]. The relationship pH−p[H] = 0.05 was observed. [OH^−^] value was calculated using a pK_w_ value of 13.921 [26]. For the variable temperature studies the following values of pK_w_ were employed at 20°C (pK_w_ = 14.126) at 30°C (pK_w_ = 13.753), at 35°C (pK_w_ = 13.660).

### 2.3. Data Processing

The calculations were obtained from ca. 100 data points in each titration using the computer program MINIQUAD-75 [27]. The stoichiometry and stability constants of the complexes formed were determined by trying various possible composition models. The model selected gave the best statistical fit and was chemically consistent with the titration data without giving any systematic drifts in the magnitudes of various residuals, as described elsewhere [27]. The fitted model was tested by comparing the experimental titration data points and the theoretical curve calculated from the values of the acid dissociation constant of the ligand and the formation constants of the corresponding complexes. The species distribution diagrams were obtained using the program SPECIES [28] under the experimental condition employed. All measurements were carried out in our laboratory in Cairo University.

## 3. Results and Discussion

### 3.1. Protonation Constants of BAPP Ligand

**Figure 1:**
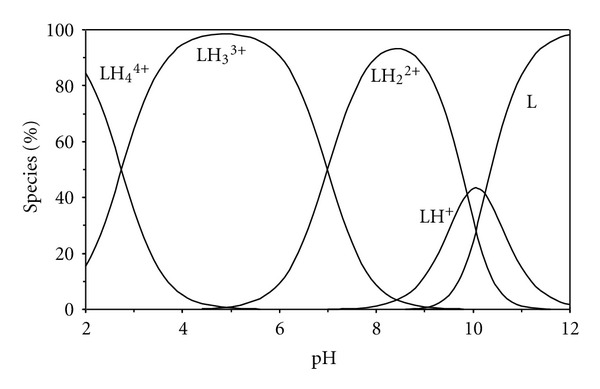
Concentration distribution of various species as a function of pH in the BAPP system (1.25 mM of BAPP).

The stoichiometric protonation constants of the investigated BAPP ligand were determined in aqueous solution at 25°C and these constants are tabulated in Table 1. The BAPP ligand studied here have four protonation constants. This is also illustrated in the species distribution of the BAPP ligand in Figure 1. In acidic solution (pH < 3), BAPP initially exists in the fully protonated form as H_4_L^+4^. On rising the pH, the species (H_4_L^+4^) lose its protons forming (H_3_L^+3^), which is the predominant species in pH range 3.0–7.0. As pH increases, the second and third protons are deprotonated forming the species (H_2_L^+2^) and (HL^+^) with maximum concentration percentage of 93% and 43% at pH 8.5 and 10, respectively. With further increase of pH, the fourth proton begins deprotonation to give the full deprotonated species (L) which is the predominant species at pH > 10.5. 

### 3.2. Binary Complexes of 1,4-Bis(3-aminopropyl)piperazine (BAPP)

The formation constants of M^II^-BAPP complexes were determined. The potentiometric titration curves for Cu^2+^, Co^2+^, Ni^2+^, and Zn^2+^ with BAPP are significantly lower than the BAPP titration curve, corresponding to formation of a complex through release of a proton. The potentiometric data of M^II^-BAPP solution mixture were fitted assuming the formation 1 : 1 species but not 1 : 2 species. The formation of the 1 : 2 complex seems to be hindered because BAPP ligand is a tetradentate ligand. The stability constants of their complexes are given in Table 2. 

### 3.3. Correlation of the Properties of Metal Ions with the Formation Constants of Mixed Ligand Complexes

**Figure 2:**
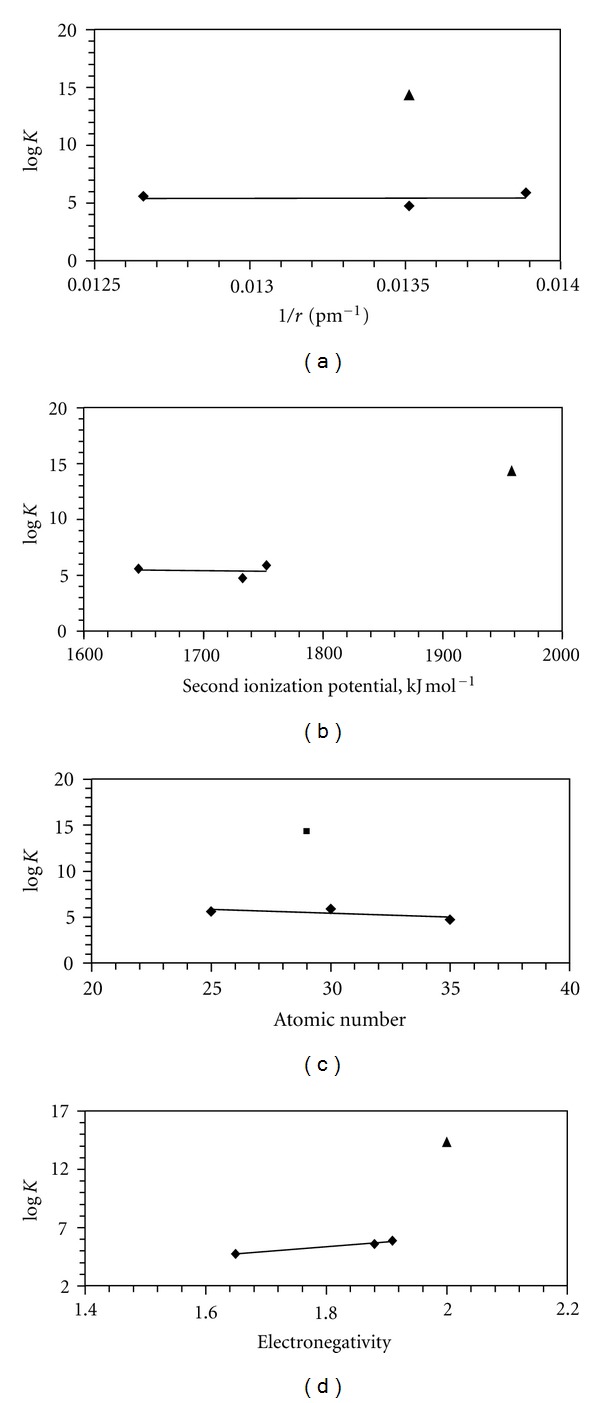
Effect of metal ion properties on the stability constants of metal complexes.

In an attempt to explain why a given ligand prefers binding to one metal rather than another, it is necessary to correlate the stability constants with the characteristic properties of the metal ions, such as the ionic radius, ionization energy, electronegativity, and the atomic number, investigated. The formation constants of M^II^-complexes of bivalent 3d transition metal ions with BAPP as given in Table 2 are in the order: Co^2+^ (log *β*_110_ = 5.59) < Ni^2+^ (log *β*_110_ = 5.88) < Cu^2+^ (log *β*_110_ = 14.26) > Zn^2+^ (log *β*_110_ = 4.74) in accordance with Irving and Williams order [29]. Here we have discussed relationships between the properties of central metal ions reported in Table 3 [30] and the stability constants of complexes. The correlation between the log⁡*K*_ML_ and the reciprocal ionic radii (1/*r*) of the studied bivalent transition metal ions was found to be almost linear (Figure 2). Also, a good linear correlation has been obtained between log⁡*K*_ML_ and the electronegativities of the metal ions under study (Figure 2). This in accordance with the fact that increasing electronegativity of the metal ions (Zn^2+^ (1.65) < Co^2+^ (1.88) < Ni^2+^ (1.91) < Cu^2+^ (2.0)) will decrease the electronegativity difference between the metal atom and the donor atom of the ligand. Thus, the metal-ligand bond would have more covalent character, which may lead to greater stability of the metal chelates. A good linear relationship has been obtained between log⁡*K*_ML_ and the second ionization potential of the bivalent metal ions under study. In general, it is noted that the stability constant of the Cu^2+^ complex is quite large compared to the other metals. The ligand field will give Cu^2+^ some extra stabilization due to tetragonal distortion of the octahedral symmetry [31, 32]. The Cu (II) complex will be further stabilized due to the Jahn-Teller effect [31]. Thus, log *K* value for the Cu^2+^-complex deviates significantly when log *K* values of metal chelates are plotted against properties of the metal ions. 

### 3.4. Species Distribution Curves

**Figure 3:**
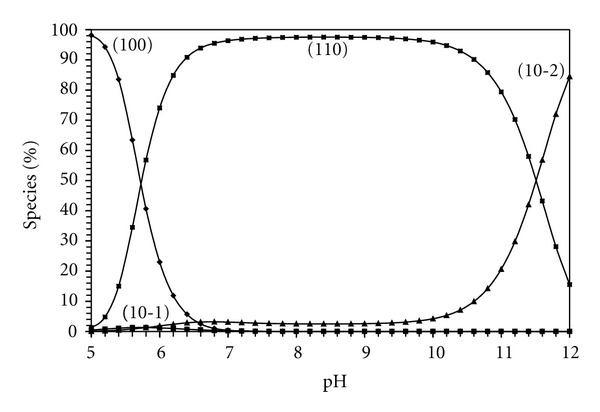
Concentration distribution of various species as a function of pH in the Zn^II^-BAPP system (1.25 mM of Zn^2+^ and BAPP).

Estimation of equilibrium concentrations of metal (II) complexes as a function of pH provides a useful picture of metal ion binding in solutions. The concentrations of metal-ligand complexes increase with increasing of pH. The species distribution pattern for Zn(BAPP)^2+^ complex, taken as a representative of metal ligand complexes, is given in Figure 3. Zn(BAPP)^2+^ complex starts to form at pH ~ 5 and reaches its maximum concentration of 98% at pH ~ 8. 

### 3.5. Effect of Temperature

**Figure 4:**
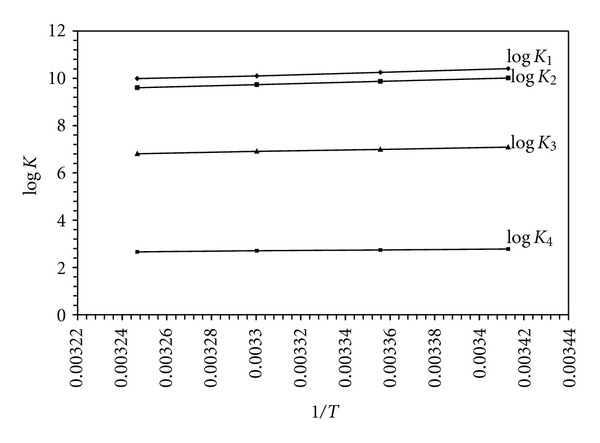
Effect of temperature on stepwise protonation constant of H_4_BAPP^+4^. Curves: log *K*_1_ corresponds to the 011 species; log *K*_2_ corresponds to 012 species; log *K*_3_ corresponds to 013 species; log *K*_4_ corresponds to 014 species.

**Figure 5:**
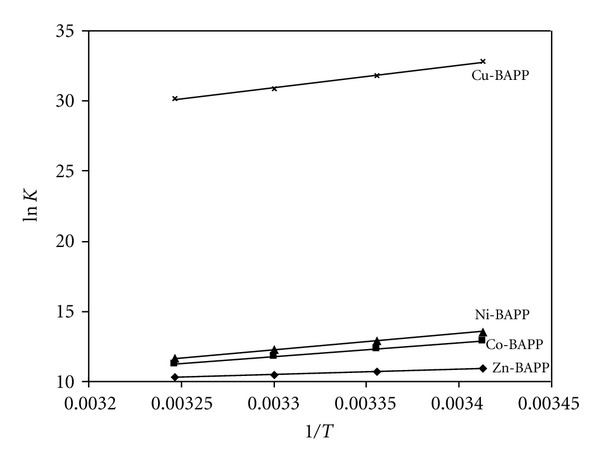
Effect of temperature on ln *K* of M^2+^ complexes with BAPP.

The values obtained for the thermodynamic parameters Δ*H* ^0^, Δ*S* ^0^, and Δ*G* ^0^, associated with the protonation of BAPP and its complex formation with M(II) species, were calculated from the temperature dependence of the data. Δ*H* ^0^ and Δ*S* ^0^ were obtained by linear least square fit of ln *K* versus 1/*T* (ln *K* = −Δ*H* ^0^/*RT* + Δ*S* ^0^/*R*) leading to an intercept Δ*S* ^0^/*R* and a slope −Δ*H* ^0^/*R*, where *K* is the equilibrium constant, (Figures 4 and 5), The main conclusions from the data can be summarized as follows.

- The protonation reactions of the *N*-site of BAPP is exothermicand of comparable Δ*H* ^0^ and Δ*S* ^0^ with a net negative Δ*G* ^0^ (Table 4). Three factors affect the protonation reactions.
  - The neutralization reaction, which is an exothermic reaction process.
  - Desolvation of ions, which is an endothermic process.
  - The change of the configuration and the arrangements of the hydrogen bonds around the free and the protonated ligands.
    - The negative Δ*S* ^0^ indicates that the total number of solvent molecules bound with the dissociated ligand is greater than that originally accompanying the undissociated form.
- The stability constants of the complexes formed at different temperatures decreased with increasing temperature, confirming that the complexation process is more favorable at lower temperatures. The thermodynamic parameters of metal complexes were calculated by a procedure similar to that used for the protonation of BAPP and the values are recorded in Table 5. It is known that the divalent metal ions exist in solution as octahedrally hydrated species [29] and the obtained values of Δ*H* ^0^ and Δ*S* ^0^ can then be considered as sum of two contributions: (a) release of H_2_O molecules and (b) metal-ligand bond formation. From these results the following conclusions can be reached.
  1. All values of Δ*G* ^0^ for complexation are negative, indicating the spontaneity of the chelation process.
  2. The negative values of Δ*H* ^0^, show that the chelation process is exothermic, indicating that the complexation reactions are favored at low temperatures.

### 3.6. Ternary Complexes Involving Zn^2+^, BAPP and Amino Acids, Peptides, or DNA Constituents

#### 3.6.1. Ternary Complex Formation Equilibria Involving Amino Acids

**Figure 6:**
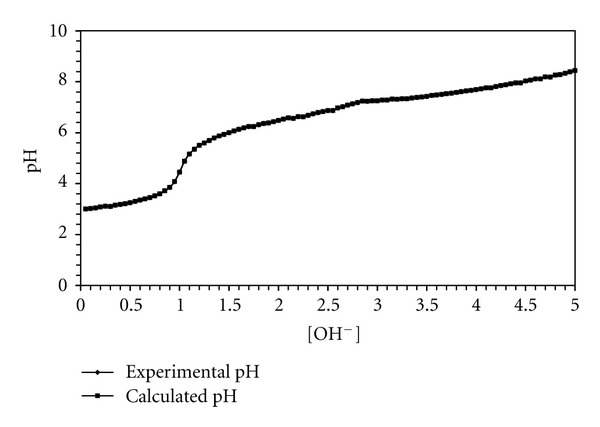
Experimental and calculated potentiometric titration curves of the Zn^II^-BAPP-Threonine system.

Ternary complex formation may proceed either through a stepwise or simultaneous mechanism depending on the chelating potential of BAPP and the other ligand (L). The formation constant of 1 : 1 Zn^II^-BAPP complex is of the same order of magnitude like Zn^II^-ligand (L) complex, (Table 6). It is reasonable to propose that in presence of both ligands, the reaction proceeds by simultaneous mechanism. This assumpation was supported by comparing the experimental potentiometric data with the theoretically calculated (simulated) curve, (Figure 6). Thus, the formation of ternary complex can be described by the following equilibrium (charges are omitted for simplicity):

(1) $$\begin{array}{c} \text{Zn}+\text{BAPP}+\text{L}⇌\text{Zn}\left(\text{BAPP}\right)\text{L} \end{array}$$

**Figure 7:**
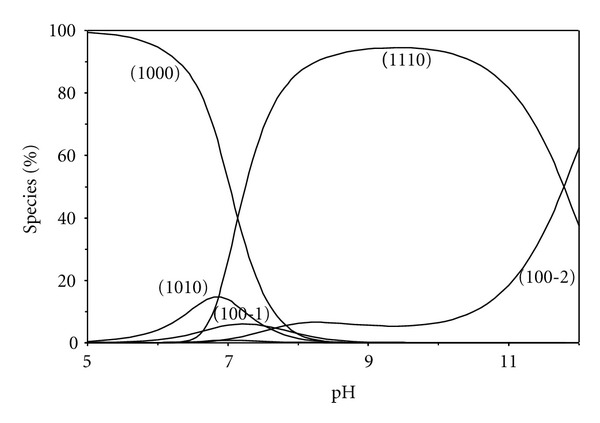
Concentration distribution of various species as a function of pH in the [Zn(BAPP)(threonine)] system (1.25 mM of Zn^2+^, BAPP, and threonine).

Figure 5 represents such a comparison for threonine system, from which it follows that the experimental data coincide with the theoretical curve. This supports the validity of the ternary complex formation model. The potentiometric data of the ternary complexes involving simple amino acids best fits assuming a complex of stoichiometric coefficient 1110 species for amino acids except for glutamic acid, ornithine, and histamine, where both 1110 and 1111 species are formed. Estimation of the concentration distribution of the various species in solution provides a useful picture of metal ion binding. The speciation diagram for threonine complex, taken as a representative amino acid, is given in Figure 7. The deprotonated species 1110 attains a maximum concentration of 99% at pH 8.8, therefore the ternary complexes of amino acids predominates in the physiological pH range. The pK_*a*_ values for the histamine complex is 9.24, being higher than that of the protonated imidazole (pK_*a*_ = 6.12), but closer to that of the protonated amino group (NH_3_^+^) in histamine ligand (pK_*a*_ = 9.85). This reveals that the proton in the protonated complex would be located mainly on the amino group. 

**Figure 8:**
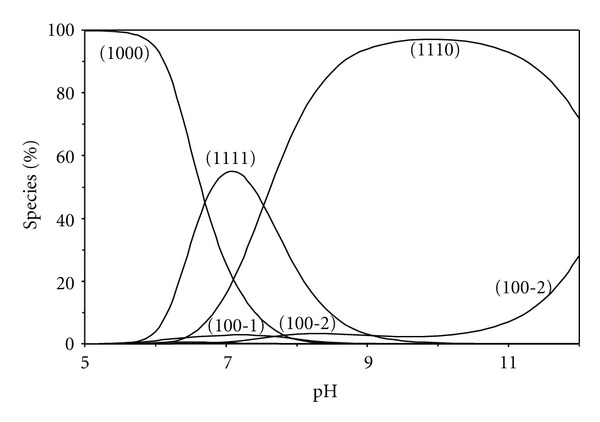
Concentration distribution of various species as a function of pH in the [Zn(BAPP)(glutamic acid)] system (1.25 mM of Zn^2+^, BAPP, and glutamic acid).

The pK_*a*_ of the protonated species of Zn(BAPP)-glutamic acid is (7.58), being higher than that of the carboxylate group (pK_*a*_ = 4.15), but near to that of the protonated amino group NH_3_^+^ (pK_*a*_ = 9.58), suggesting that the proton in the protonated complex would be located mainly on the amino group, considering the increase in acidity due to complex formation. The species distribution for glutamic acid complex, taken as a representative, is given in Figure 8. The protonated species 1111 complex predominates with formation percentage of (55% at pH ~7.0); the deprotonated species 1110 complex reaches the maximum concentration of 97% at pH ~9.8. Therefore the species 1111 complex predominates in the physiological pH range.

Ornithine is *α*-amino acid having an extra amino group. Ornithine forms protonated complex 1111 species and the pK_*a*_ value amounts to 7.10. This is fairly comparable with the acid protonation constant of the *δ*-amino group, considering the increase in acidity due to complex formation. 

#### 3.6.2. Ternary Complex Formation Equilibria Involving Amides

Ternary complex formation of amides proceeds also through simultaneous mechanism (Table 7). The potentiometric data reported for the peptides, serine, and threonine complexes reveals the formation of Zn(BAPP)(L) species rather than the Zn(BAPP)LH_−1_ species which supports the view that induced ionization of peptide hydrogen would be unfavoured. This finding is in agreement with previous investigation carried out on the Zn(II)-diethylenetriamine-peptide and Zn(II)-nitrilo-tris(methyl phosphonic acid)-peptide systems [33].

 The glutamine complex is more stable than the glycinamide complex, presumably due to the fact that glutamine carries a negative charge, whereas glycinamide is neutral. The electrostatic interaction between the glutaminate and the positively charged Zn(II) complex would result in a lowering of the free energy of formation.

**Figure 9:**
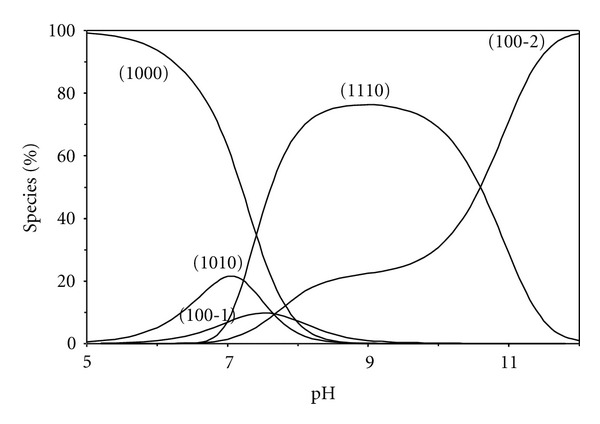
Concentration distribution of various species as a function of pH in the [Zn(BAPP)(glycinamide)] system (1.25 mM of Zn^2+^, BAPP, and glycinamide).

The speciation diagram of glycinamide complex as a representative of amides is given in Figure 9. The mixed ligand species [Zn(BAPP)L] (1110) starts to form at pH~6.8 and with increasing pH, its concentration increases reaching the maximum of 76% at pH ~ 9. The species with concentration percentage less than 5% were neglected in the concentration distribution plot for clarity.

#### 3.6.3. Ternary Complex Formation Equilibria Involving DNA Units

**Figure 10:**
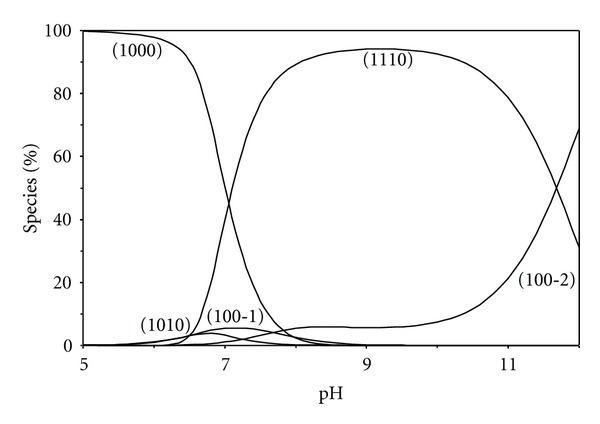
Concentration distribution of various species as a function of pH in the [Zn(BAPP)(inosine)] system (1.25 mM of Zn^2+^, BAPP, and inosine).

Inosine is slightly more acidic than the pyrimidinic species (thymine and thymidine). This can be related to the existence of the anion form of purinic derivatives in a higher number of resonance forms due to the presence of two condensed rings in this ligand (inosine). Based on the existing data, thymine and thymidine ligate in the deprotonated form as monoanions, through N3, and they do not form protonated complexes. As a result of the high pK_*a*_ values of pyrimidines (pK_*a*_ *≈* 9) and the fact that they are monodentates, the complexes are formed only above pH 6, supporting the view that the negatively charged nitrogen donors of pyrimidine bases are important binding sites in the neutral and slightly basic pH ranges. The purines like inosine have two metal ion binding centres N1 and N7 nitrogens. Inosine may become protonated at N(7) with formation of [N(1)H–N(7)H] monocations. In the present study, the pK_*a*_ of N(1)H was only determined since the pK_*a*_ of N(7)H is too low to be detected by the potentiometric technique. In the acidic pH-range, N1 remains protonated, while the metal ion is attached to N7. The gradual change from N7-binding to N1-binding with increasing pH has been rather extensively documented by nuclear magnetic resonance (NMR) [34] and electron paramagnetic resonance (EPR) [35] spectroscopic measurements. Consequently, it is proposed that N(1) serves as a coordination site in the mixed ligand complexes of inosine at higher pH values. The speciation of inosine complex is presented in Figure 10, where the species distribution of the complexes is plotted as a function of pH. The complex [Zn(BAPP)L] (1110) reaching a maximum concentration of 94% at pH 9.

### 3.7. Comparison of the Stability Constant of the Ternary Complexes with Those of the Binary Complexes

One of the most important parameters generally used for indicating the stabilization of the mixed complexes with respect to the binary ones namely is Δlog *K*. It has been widely accepted and used for many years [36] and the advantages in using Δlog K in comparing stabilities of ternary and binary complexes have been reviewed. Δlog_10_ *K*, the difference between the stabilities of the binary and mixed complexes, expresses the effect of the bounded primary ligand towards an incoming secondary ligand (L). One expects to obtain negative values for Δlog *K* (Table 7), since more coordination positions are available for the bonding of ligand (L) in the binary than in the ternary complexes. This indicates that the secondary ligand (L) amino acid, peptide, or DNA form more stable complexes with zinc (II) ion alone than with Zn^II^-BAPP complex. The Δ log *K* value for deprotonated ternary complexes is given by

(2) $$\begin{array}{c} \text{Zn}\left(\text{BAPP}\right)+\text{Zn}\left(\text{L}\right)⇌\text{Zn}\left(\text{BAPP}\right)\text{L}+\text{Zn} \end{array}$$

(3) Δlog⁡ KZn(BAPP)L=log⁡ βZn(BAPP)(L)−log⁡ βZn(BAPP) −log⁡ βZn(L).

All values of Δlog *K* for the ternary complexes studied in this paper are listed in Table 7. It is of interest to note that for all the systems listed in Table 7, the values for Δlog *K* are positive; that is, equilibrium 6 is on its right side. According to Sigel [36, 37], the relative stability of a ternary complex Zn(BAPP)L(1110) compared to its binary complex Zn(BAPP) (1100) or Zn(L) (1010) can be expressed quantitatively by (2) and (3). Zn-BAPP (1 : 1) has fewer coordination sites than the aquatic Zn^2+*‏*^ ion complex. Consequently, the secondary ligands (L) are expected to bind to the Zn-BAPP complex with a smaller stability constant than with an aquatic metal ion. Therefore, Δlog_10_ *K* should be negative and generally have a value between −0.5 and −2.0 [36, 38] depending on the geometry of the complex. For Zn*‏* ^2+^ ions, positive values of Δlog_10_ *K* may be considered as evidence for the occurrence of enhanced stabilities. This may be explained on the premise that: a sort of hydrophobic intramolecular interaction possibly occurred between the hydrophobic moiety of BAPP and the noncoordinating hydrophobic side chain of amino acids in aqueous solutions. Such a hydrophobic interaction has been reported in literature [36, 39–41]. 

## 4. Conclusions

The present investigation describes the formation equilibria of Zn(II) complexes involving BAPP and some ligands of biological significance. In combination of stability constants data of such Zn^II^ complexes with amino acids, peptides, or DNA constituents, it would be possible to calculate the equilibrium distribution of the metal species in biological fluids where all types of ligands are present simultaneously. This would form a clear basis for understanding the mode of action of such metal species under physiological conditions. From the above results, it may be concluded that ternary complex formation of amino acids and peptides proceeds through simultaneous mechanism. The mixed ligand complexes are formed in the physiological pH range, indicating this interaction. The positive value of Δlog *K* is attributed to the extra stability of the ternary complexes.

## Figures and Tables

**Table 1:** Protonation constants of (BAPP) in aqueous solution at different temperatures (at 0.01 M ionic strength).

Temp. (°C)	log⁡*K*_1_^*H*^	log⁡*K*_2_^*H*^	log⁡*K*_3_^*H*^	log⁡*K*_4_^*H*^
20	10.41	10.01	7.09	2.78
25	10.25	9.87	6.99	2.74
30	10.1	9.73	6.91	2.71
35	9.99	9.6	6.81	2.66

log⁡*K*_1_^*H*^, log⁡*K*_2_^*H*^, log⁡*K*_3_^*H*^ and log⁡*K*_4_^*H*^ are the stepwise protonation constants.

**Table 2:** Stepwise stability constants for the complexation of (BAPP) with 3d divalent metal ions in aqueous solution at 0.1 M NaNO_3_ at different temperatures.

Cations	log⁡*K* ^a^			
	20°C	25°C	30°C	35°C
ZnL	4.74 (0.06)	4.64 (0.08)	4.55 (0.08)	4.47 (0.07)
CoL	5.59 (0.02)	5.35 (0.09)	5.12 (0.06)	4.88 (0.10)
NiL	5.88 (0.04)	5.61 (0.02)	5.33 (0.03)	5.06 (0.02)
CuL	14.26 (0.02)	13.81 (0.01)	13.42 (0.05)	13.09 (0.07)

^a^Standard deviations are given in parentheses, L: BAPP.

**Table 3:** Atomic number, ionic radius, electronegativity, and ionization potential of the investigated bivalent metal ion^a^.

Metal ion	Co^2+^	Ni^2^	Cu^2+^	Zn^2+^
Atomic number	27	28	29	30
Ionic radius (pm)	79	72	71	74
Electronegativity	1.88	1.91	2.00	1.65
Second ionization energy (kJ/mol)	1646	1753	1958	1733

^a^Values from [30].

**Table 4:** Thermodynamic parameters for the stepwise protonation of (BAPP) in aqueous solution (at 0.01 M ionic strength)^a^.

Equilibrium^b^	Δ*H*°	Δ*S*°	Δ*G*°
	kJMol^−1^	JK^−1^ Mol^−1^	kJMol^−1^
(1) L + H^+^ *⇌* LH^+^log⁡*K*_1_^*H*^	−48.8 (0.8)	32.7 (0.7)	−58.5 (0.8)
(2) LH^+^ + H^+^ *⇌* LH_2_^2+^log⁡*K*_2_^*H*^	−47.4 (0.7)	30.1 (0.6)	−56.3 (0.8)
(3) LH_2_^2+^ + H^+^ *⇌* LH_3_^3+^log⁡*K*_3_^*H*^	−31.8 (0.6)	27.3 (0.5)	−39.9 (0.8)
(4) LH_3_^3+^ + H^+^ *⇌* LH_4_^4+^log⁡*K*_4_^*H*^	−13.5 (0.3)	7.31 (0.12)	−15.6 (0.3)

^a^Standard deviations are given in parentheses; ^b^ must be under Table 4 refer to L, where L denotes 1,4-bis(3-aminopropyl)-piperazine (BAPP)

**Table 5:** Thermodynamic parameters for ML complexes of (BAPP) with 3d divalent metal ions in aqueous solution at 0.1 M NaNO_3 _ at different temperatures^a^.

Equilibrium	Δ*H*°	Δ*S*°	Δ*G*°
	kJMol^−1^	JK^−1^ Mol^−1^	kJMol^−1^
(1) Zn^2+^ + L *⇌* ZnL^2+^	−31.1 (0.4)	−15.5 (0.7)	−26.5 (0.8)
(2) Co^2+^ + L *⇌* CoL^2+^	−81.5 (0.8)	−171.2 (1.2)	−30.5 (0.4)
(3) Ni^2+^ + L *⇌* NiL^2+^	−94.7 (0.9)	−210.4 (1.9)	−32.0 (0.4)
(4) Cu^2+^ + L *⇌* CuL^2+^	−134.5 (1.1)	−186.7 (1.5)	−78.9 (0.9)

^a^Standard deviations are given in parentheses; L: BAPP.

**Table 6:** Stability constants of the ternary species in the Zn^II^-BAPP-amino acid, amide, or DNA constituent systems and proton-association constants and their binary stability constants.

*l* *p* *q* *r* ^a^		log⁡*β* ^b^	
	OH^−^	BAPP	
1 0 0-1	−7.96 (0.01)	—	
1 0 0-2	−15.64 (0.01)	—	
0 1 0 1	—	10.25 (0.01)	
0 1 0 2	—	20.12 (0.02)	
0 1 0 3	—	27.11 (0.03)	
0 1 0 4	—	30.26 (0.01)	
1 1 0 0	—	4.64 (0.03)	

	Alanine	Threonine	Serine

0 0 1 1	9.69 (0.01)	9.06 (0.01)	9.14 (0.01)
0 0 1 2	11.88 (0.02)	11.03 (0.02)	11.40 (0.01)
1 0 1 0	4.58 (0.03)	4.63 (0.09)	4.60 (0.09)
1 0 2 0	8.71 (0.06)	8.62 (0.08)	8.45 (0.08)
1 1 1 0	14.91 (0.01)	14.36 (0.08)	16.86 (0.08)

	Ornithine	Glutamic acid	Histamine·2HCl

0 0 1 1	10.58 (0.00)	9.58 (0.01)	9.85 (0.01)
0 0 1 2	19.43 (0.02)	13.73 (0.01)	15.97 (0.01)
0 0 1 3	21.39 (0.02)	—	—
1 0 1 0	3.75 (0.09)	4.12 (0.09)	5.22 (0.02)
1 0 2 0	6.44 (0.05)	7.65 (0.05)	10.18 (0.05)
1 1 1 0	17.71 (0.06)	15.68 (0.10)	12.39 (0.10)
1 1 1 1	24.81 (0.06)	23.21 (0.10)	21.63 (0.07)

	Glycinamide	Glutamine	

0 0 1 1	7.60 (0.01)	8.95 (0.01)	
1 0 1 0	3.28 (0.07)	4.12 (0.06)	
1 1 1 0	12.47 (0.06)	15.41 (0.08)	

	Inosine	Thymine	Thymidine

0 0 1 1	8.55 (0.02)	9.58 (0.02)	9.50 (0.01)
1 0 1 0	3.52 (0.10)	4.82 (0.08)	4.47 (0.08)
1 1 1 0	14.15 (0.10)	15.89 (0.10)	15.72 (0.09)
1 1 1 1	20.38 (0.10)		

^a^ *l*, *p*, *q* and *r* are stoichiometric coefficients corresponding to Zn^II^, BAPP, other ligand, and H^+^, respectively.

^b^Standard deviations are given in parentheses sum of square of residuals are less than 5E-7.

**Table 7:** Δlog⁡*K* = log⁡*β*_1110_ − (  log⁡*β*_1100_ + log⁡*β*_1010_).

	Δlog⁡*K*
Alanine	5.69
Threonine	5.09
Serine	7.62
Ornithine	9.32
Glutamic acid	6.92
Histamine·2HCl	2.53
Glycinamide	4.55
Glutamine	6.65
Inosine	5.99
Thymidine	6.61
Thymine	6.43
